# Increased measles and rubella seroprevalence in children using residual blood samples from health facilities and household serosurveys after supplementary immunization activities in two districts in India

**DOI:** 10.1017/S0950268824001353

**Published:** 2024-11-18

**Authors:** Christine Prosperi, Alvira Z. Hasan, Amy K. Winter, Itta Krishna Chaaithanya, Neha R. Salvi, Sandeep Sharma, Avi Kumar Bansal, Sanjay L. Chauhan, Ragini N. Kulkarni, Abhishek Lachyan, Poonam Gawali, Mitali Kapoor, Arpit Kumar Shrivastava, Saurabh K. Chonker, Vaishali Bhatt, Ojas Kaduskar, Gururaj Rao Deshpande, Ignacio Esteban, R. Sabarinathan, Velusamy Saravana Kumar, Shaun A. Truelove, Muthusamy Santhosh Kumar, Jeromie W. Vivian Thangaraj, Lucky Sangal, Sanjay M. Mehendale, Gajanan N. Sapkal, Nivedita Gupta, Kyla Hayford, William J. Moss, Manoj V. Murhekar

**Affiliations:** 1 International Vaccine Access Center, Department of International Health, Johns Hopkins Bloomberg School of Public Health, Baltimore, Maryland, USA; 2 Department of Epidemiology, Johns Hopkins Bloomberg School of Public Health, Baltimore, MD, USA; 3 Department of Health Research, Model Rural Health Research Unit, Dahanu, Maharashtra, India; 4 Indian Council of Medical Research (ICMR)-National JALMA Institute for Leprosy & Other Mycobacterial Diseases, Agra, India; 5 Indian Council of Medical Research - National Institute for Research in Reproductive and Child Health (NIRRCH), Mumbai, India; 6 Department of Health Research, Model Rural Health Research Unit, Kanpur, Uttar Pradesh, India; 7 Diagnostic Virology Group, Indian Council of Medical Research-National Institute of Virology, Pune, Maharashtra, India; 8 Indian Council of Medical Research (ICMR)-National Institute of Epidemiology, Chennai, India; 9 World Health Organization, Southeast Asia Region Office, New Delhi, India; 10 PD Hinduja Hospital and Medical Research Centre, Mumbai, India; 11 Division of Epidemiology and Communicable Diseases, Indian Council of Medical Research, New Delhi, India

**Keywords:** measles (rubeola), rubella, serology, vaccines

## Abstract

Residual blood specimens provide a sample repository that could be analyzed to estimate and track changes in seroprevalence with fewer resources than household-based surveys. We conducted parallel facility and community-based cross-sectional serological surveys in two districts in India, Kanpur Nagar District, Uttar Pradesh, and Palghar District, Maharashtra, before and after a measles-rubella supplemental immunization activity (MR-SIA) from 2018 to 2019. Anonymized residual specimens from children 9 months to younger than 15 years of age were collected from public and private diagnostic laboratories and public hospitals and tested for IgG antibodies to measles and rubella viruses. Significant increases in seroprevalence were observed following the MR SIA using the facility-based specimens. Younger children whose specimens were tested at a public facility in Kanpur Nagar District had significantly lower rubella seroprevalence prior to the SIA compared to those attending a private hospital, but this difference was not observed following the SIA. Similar increases in rubella seroprevalence were observed in facility-based and community-based serosurveys following the MR SIA, but trends in measles seroprevalence were inconsistent between the two specimen sources. Despite challenges with representativeness and limited metadata, residual specimens can be useful in estimating seroprevalence and assessing trends through facility-based sentinel surveillance.

## Introduction

Serological surveillance for vaccine-preventable diseases (VPDs), such as measles and rubella, provides direct evidence of immunity gaps and changes in seroprevalence following immunization activities [1]. Evidence from serological surveys can guide immunization programmes, for example, on expanding the age range of a vaccination campaign or identifying geographical areas such as districts or states for targeted vaccination activities. Rigorously conducted representative household surveys employing probability-based sampling and blood collection can generate high-quality seroprevalence estimates. However, these surveys can be logistically challenging, expensive, and require participants to voluntarily provide blood specimens [2, 3]. While these surveys are often considered the ‘gold standard’, biases may be introduced due to incomplete or incorrect sampling frames or participant non-response [4–6].

Residual samples of blood collected for other purposes provide an alternative specimen repository that could be analyzed to estimate and track changes in seroprevalence and identify immunity gaps. Residual blood specimens include those collected for clinical purposes at a health facility or laboratory, from blood donations, or those available in a biorepository from other studies, surveys, or surveillance systems [7–10]. However, serosurveys that use residual specimens have several limitations, including selection bias and limited metadata linked to the specimens. Consequently, the tradeoffs between bias, completeness of data, ease of collection, and cost need to be weighed carefully.

Few prior studies have explored concurrent comparisons between community-based and residual specimen serosurveys. With the surge in use of residual specimens to study SARS-CoV-2 seroprevalence and trends, there was increased interest in comparing seroprevalence estimates with those derived from community-based specimens [11]. Two studies conducted in urban U.S. settings (Atlanta, Georgia and Denver, Colorado) in 2020 compared SARS-CoV-2 estimates from specimens collected at commercial laboratories and concurrent community-based serosurveys and both found similar results between the two specimen sources [12, 13]. In each study, the two specimen sources were collected in overlapping geographic areas and time periods, and the results were weighted to align with the underlying population age and sex distributions. However, both studies highlighted the lack of race and ethnicity data for the residual specimens as a limitation. An earlier study in Australia compared seroprevalence estimates of measles, mumps, rubella, varicella, and hepatitis B obtained using residual specimens from the national residual sera bank that received specimens from diagnostic laboratories with those from a school-based cluster survey. No significant differences in seroprevalence were found except for rubella, which they attributed to the use of different assays [14]. The cost of collecting and storing residual specimens was approximately seven times less than the cost of specimens collected during the school-based, random cluster sample. All three head-to-head comparisons were conducted in high-income settings, which may have different biases in using residual specimens.

We conducted serosurveys using specimens collected at health facilities and diagnostic laboratories (elsewhere referred to as “residual specimens”) in Palghar District, Maharashtra, and Kanpur Nagar District, Uttar Pradesh, before and after a measles-rubella supplemental immunization activity (MR-SIA) conducted from 2018 to 2019 in India targeting children 9 months to younger than 15 years of age [15, 16]. India follows a two-dose MR vaccine strategy, with the first dose given between 9–12 months and the second between 16 – 24 months of age [17]. The goal of the study was to assess if estimates of measles and rubella serosurveillance using residual specimens can be used to monitor changes in seroprevalence following the MR-SIA and identify age-specific immunity gaps. We also explored how the changes in seroprevalence using residual specimens compared to those using specimens collected from concurrent community-based serosurveys [18].

## Methods

### Survey setting

**Figure 1.**
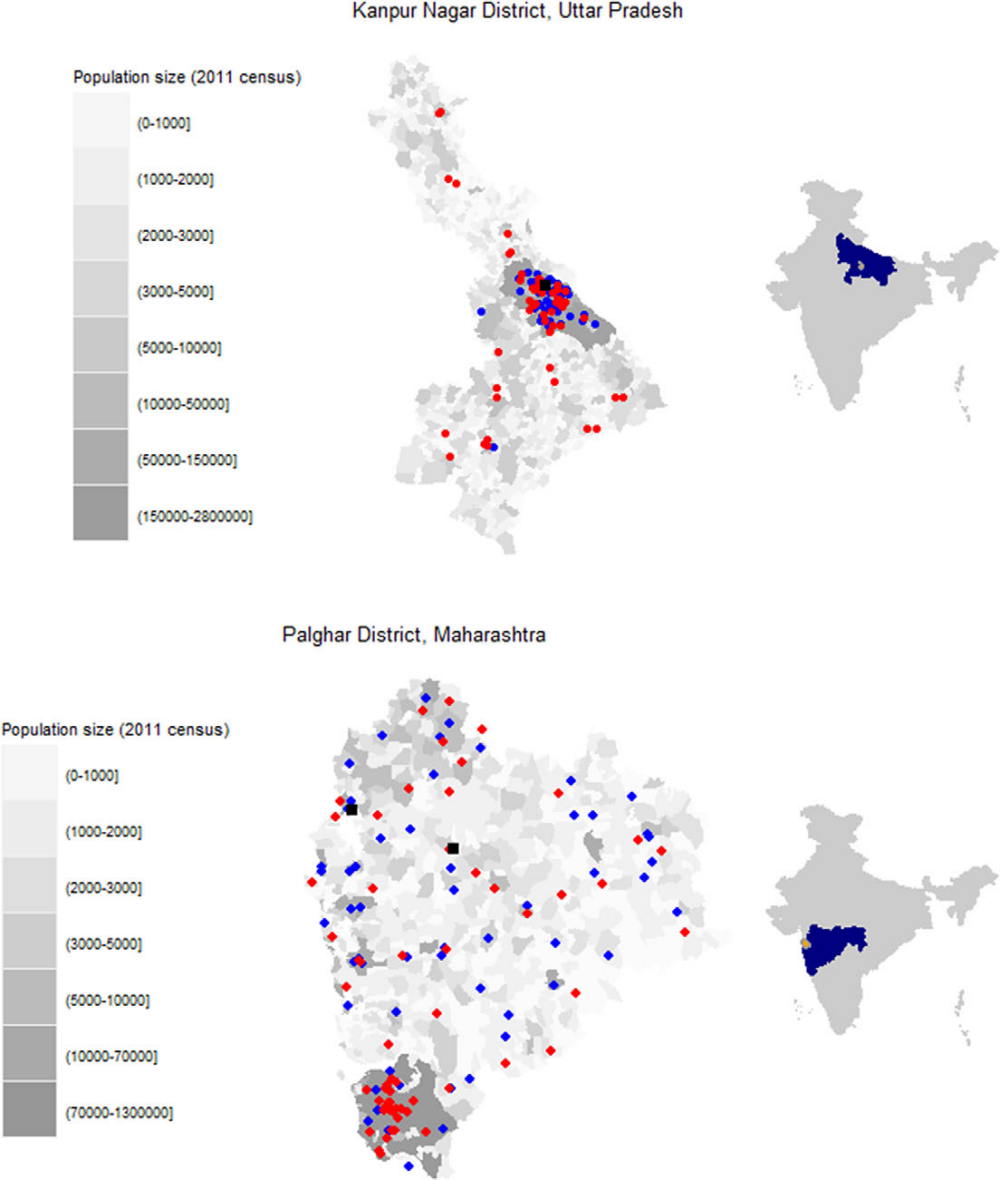
Location of facilities where residual specimens were collected and survey clusters for community-based serosurvey. (a) Kanpur Nagar District, Uttar Pradesh. (b) Palghar District, Maharashtra. Legend: Grayscale corresponds to the population size of each enumeration area based on the 2011 census estimates. Red circles indicate community-based survey clusters. Blue circles indicate the originating facilities for specimens received at Paliwal Diagnostics (Kanpur Nagar District) or Hind Laboratory (Palghar District). Black squares indicate fixed point residual specimen collection at G.S.V.M. Medical College (Kanpur Nagar District) or the subdistrict hospitals in Dahanu and Kasa (Palghar District). Right side maps indicate the state (blue) and district (orange) locations within the country.

Potential facilities were identified within each district based on population size, public versus private hospital usage, and rural versus urban distribution in the district. Of the facilities selected, only some were willing to participate. Specimens were collected from four facilities in Palghar District, including two facilities of a public diagnostic laboratory (Hind Lab-Dahanu and Hind Lab-Jawhar) and two public hospitals (subdistrict hospitals in Dahanu and Kasa; Figure 1, Supplementary Table S1). In Kanpur Nagar District, two facilities were selected based on input from the local study team with the goal of identifying facilities that were large and most representative of the district. These included one private diagnostic laboratory (Paliwal Diagnostics) and one public hospital (Ganesh Shankar Vidyarthi Memorial [GSVM] Medical College) (Figure 1, Supplementary Table S1). Both facilities served the entire district, although they were in the Kanpur Nagar metropolitan area. Additional details on the facilities are included in the supplemental appendix.

### Residual specimens

Convenience specimens of anonymized residual sera were collected from patients in two age groups (younger than 5 years, 5 to younger than 15 years) at the participating facilities and laboratories. Specimens were collected from November 2018 to July 2019 during discrete periods before, during, and after the MR-SIA (Supplemental Table S2). The number of pre-SIA specimens was limited due to the timing of project initiation relative to the SIA.

In each district, the serosurvey was implemented by the local Model Rural Health Research Unit (MRHRU), which was established by the Department of Health Research (DHR) with a mandate to work on local disease burdens [19]. Information on specimen volume, specimen collection procedures, and specimen flow was obtained through observations and discussions with facility staff and used to guide procedures for residual specimen collection. The facility was requested to retain all residual specimens after testing. Study staff visited the facility to identify and collect eligible specimens and abstract key data points. Depending on the timing related to facility procedures and space available to store specimens at 2-8 °C, the specimens were collected by study staff either at the end of the day, the following morning, or within 96 h of collection from the patient. All specimens collected were anonymized and re-labelled with a study identification number. Data on age, sex, and date of specimen collection were obtained from the laboratory paper register or electronic database. Other data, such as the patient’s residence (ward or village name) and ward where the patient was seen at the hospital (outpatient, inpatient) were also collected from the laboratory register or database, if available. For residual specimens collected from diagnostic laboratories, information about the originating facility was also collected. Specimens were excluded if age was not available, if received by the study staff more than 96 h of collection from the patient, or if there was no visible serum in the tube. Study staff rotated the days when specimens were collected at a given facility (e.g., SDH Dahanu on Monday, Hind Dahanu on Tuesday).

The residual specimens were centrifuged at the facility (3,000 rpm for 10 min) and stored at 4-8 °C in cold boxes until transported to the central laboratory at the MRHRUs for processing and storage. At the MRHRU laboratory, sera were aliquoted and stored at -20 °C within 24 h of collection and later transported by plane or vehicle to the Indian Council of Medical Research (ICMR)-National Institute of Virology, Pune, in a cold box with dry ice for testing.

### Community-based specimens

Community-based cross-sectional serosurveys were conducted in both districts before and after the MR-SIA by the MRHRU teams involved in the residual specimen collection as previously described [18]. The sampling frame was derived to estimate measles and rubella seroprevalence at the district level. Briefly, thirty villages or wards were selected from each district based on the 2011 census using probability proportional to size systematic sampling (Figure 1). One census enumeration block (CEB) was randomly selected from the list of CEBs in each village or ward. Following enumeration of all individuals in the CEB, thirteen individuals were randomly selected from each of two age groups: children nine months to younger than 5 years and children aged 5 to younger than 15 years. A venous blood sample and information on sociodemographic characteristics and vaccination history were collected after obtaining written parental permission, informed consent, or assent. The blood specimens were processed to sera at the MRHRU laboratory and then transported to the ICMR-National Institute of Virology, Pune, under cold chain, as for the residual specimens.

### Specimen testing

Sera were tested for IgG antibodies to measles and rubella viruses using Euroimmun quantitative IgG enzyme immunoassays (Euroimmun AG, Lübeck, Germany; measles product code: EI 2610-9601G; rubella: EI 2590-9601G) following the manufacturer’s instructions. Specimens from both surveys were tested using the same assay kits, but testing was done at different times with different lots. One of the measles kit calibrators changed, which had an impact on lower quantitative results around the threshold. A linear correction derived from a lot-to-lot comparison was applied to the residual specimen estimates to enable comparison between the two specimen sources as previously described [18].

### Statistical analysis

A sample size of at least 375 residual sera per age group and facility were needed to detect ≥10% absolute difference in measles and rubella seroprevalence after the SIA.

This analysis is limited to residual and community-based specimens collected from children aged 9 months to younger than 15 years collected before and after the MR-SIA. Seroprevalence estimates for IgG antibodies to measles and rubella viruses were estimated with 95% confidence intervals. We explored differences in seroprevalence by sex and facility type (in Kanpur Nagar District only) with descriptive summaries and logistic regression, adjusted for age in years. For analyses using both residual and community-based serosurveys, residual specimens were age-standardized to the pre-SIA community age distribution from the same district. The community-based serosurvey estimates were calculated using sampling weights based on survey design and accounting for non-response. In Palghar District, Maharashtra, community-based analyses were restricted to children 1 year and older due to a lack of residual specimens for children 9 months to 1 year. Bonferroni-adjusted p-values were used to account for multiple comparisons.

Sensitivity analyses were conducted by geographically subsetting the community-based serosurvey data to align more closely with the location of the facilities where residual specimens were collected. To assess potential biases introduced by the geographic distribution of the residual specimens, we evaluated measles and rubella seroprevalence spatial autocorrelation for the community samples. Specifically, we estimated global Moran’s I statistic over 1,000 permutations with the R package spdep (version 1.2–5). To calculate Moran’s I, we created distance-based spatial weight matrices for both districts using a fixed distance value based on the geographic extent of the spatial results and the minimal distance needed for all clusters to be included in the analysis (13 and 16 km for Palghar and Kanpur Nagar Districts, respectively, Supplementary Figure S1). Analyses were performed using R (version 3.6.1).

### Ethical considerations

The Institutional Ethics Committees of ICMR-National Institute of Epidemiology, Chennai, India, Johns Hopkins Bloomberg School of Public Health, Baltimore, USA, and study sites approved the protocols for both community-based and residual specimen collection. For the residual serosurvey there was no interaction with human subjects, and all specimens were deidentified. For the community-based serosurvey written consent from parents of children aged between 9 months and younger than 15 years, oral assent from children aged between 7 and younger than 12 years, and written assent from children 12 to younger than 15 years were obtained before participation in the survey.

## Results

### Description of facility-based residual specimens

A total of 611 residual specimens were collected from children aged 9 months to 15 years prior to the SIA, and 2,198 residual specimens were collected after the SIA (Table 1). Most specimens were collected from children aged 5 to 15 years. To simplify procedures at the facility, residual specimens were collected from all children younger than 5 years, but those from children below 9 months of age were excluded for this analysis to allow for comparability with the community survey. In Kanpur Nagar District, approximately 60% of the pre-SIA specimens were collected from the private facility, but this imbalance was less in the post-SIA period. There was no difference in the age-group distribution by period for specimens collected at the public facility in Kanpur Nagar; however, the post-SIA private facility specimens were from significantly younger children than those collected pre-SIA (41.0% 9 months to <5 years post-SIA vs. 27.2% pre-SIA, Supplementary Table S3).

**Table 1.** Characteristics of pediatric specimens collected at health facilities before and after the MR supplemental immunization activity in Kanpur Nagar District, Uttar Pradesh and Palghar District, Maharashtra

	Kanpur Nagar, Uttar Pradesh		Palghar, Maharashtra	
	Pre-SIA n (column %)	Post-SIA n (column %)	Pre-SIA n (column %)	Post-SIA n (column %)
Total	398	1,360	213	838
Age group				
9 months - <5 years	106 (26.3)	481 (35.4)	53 (24.9)	310 (37.0)
5- < 15 years	292 (73.4)	879 (64.6)	160 (75.1)	528 (63.0)
Sex				
Female	164 (41.2)	536 (39.4)	104 (48.8)	371 (44.3)
Male	234 (58.8)	824 (60.6)	109 (51.2)	467 (55.7)
Facility type				
Public	166 (41.7)	655 (48.2)	–	–
Private	232 (58.3)	705 (51.8)	–	–
Originating facility type				
Subdistrict hospital	–	–	119 (55.9)	366 (43.7)
Rural hospital	–	–	35 (16.4)	279 (33.3)
Primary health center	–	–	59 (27.7)	193 (23.0)

Note: Restricted to specimens 9 months and older with available EIA results. Pre-SIA collection period was from 5 to 27 November 2018. Post-SIA collection period was substantially longer than the pre-SIA period, lasting from 6 March 2019 to 31 July 2019.

Abbreviation: SIA, supplemental immunization activity.

### Measles and rubella seroprevalence using facility-based specimens

**Figure 2.**
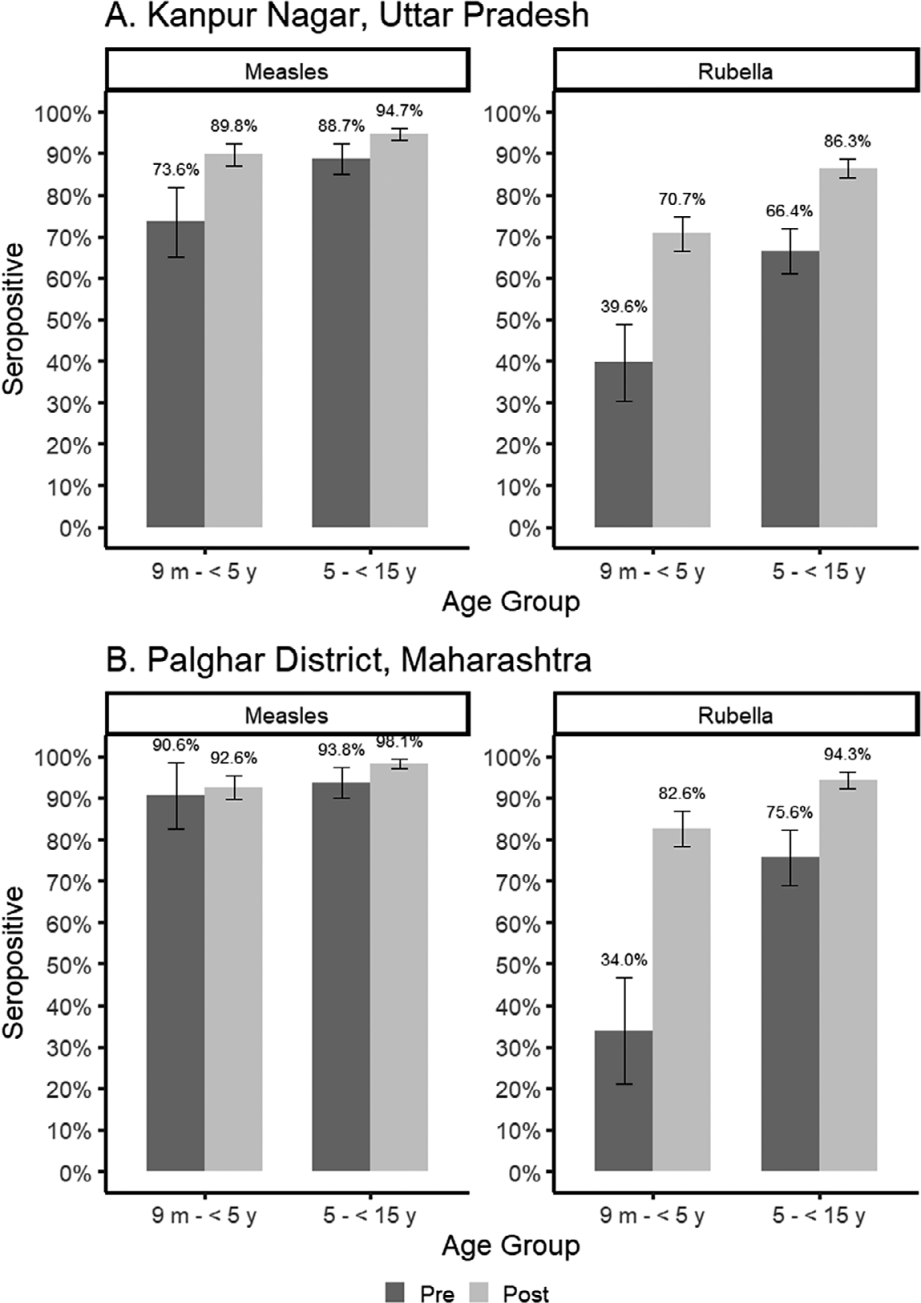
Measles and rubella seroprevalence before and after the measles-rubella supplemental immunization activity among children 9 months to <15 years seen at health facilities, by district.

A significant increase in measles and rubella seroprevalence was observed for children 9 months to 5 years and 5 to 15 years of age using the residual specimens in Kanpur Nagar District following the SIA (Figure 2, Supplementary Table S4), particularly for the younger age group (measles seroprevalence [95% CI]: 73.6 [65.2, 82.0] vs. 89.8 [87.1, 92.5]; rubella seroprevalence [95% CI]: 39.6 [30.3, 48.9] vs. 70.7 [66.6, 74.8]). However, rubella seroprevalence was below 90% for both age groups after the SIA (9 months to younger than 5 years: 70.7%, 5 to 15 years: 86.3%).

In Palghar District, no increase in measles seroprevalence was observed following the SIA for the younger age group in Palghar District (90.6 [82.7, 98.4] vs. 92.6 [89.7, 95.5]), although pre-SIA seroprevalence was high. A significant increase in measles seroprevalence was observed for the older age group and in rubella seroprevalence for both age groups. No difference in seroprevalence was observed by sex for either period or age group in either district (Supplementary Table S5).

### Measles and rubella seroprevalence by facility type

**Figure 3.**
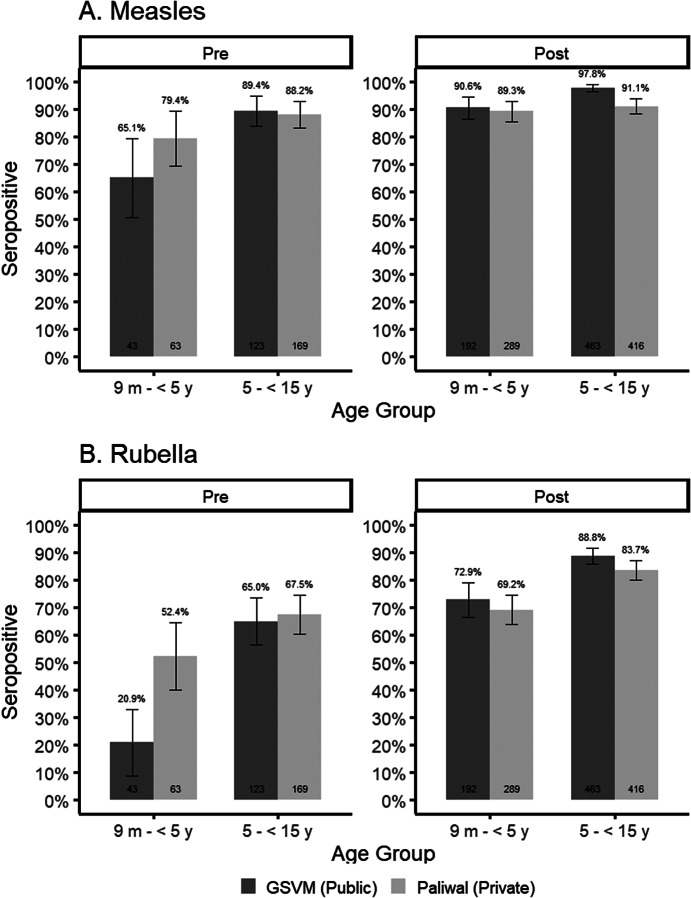
Measles and rubella seroprevalence before and after the measles-rubella supplemental immunization activity among children 9 months to <15 years seen at health facilities in Kanpur Nagar District Uttar Pradesh, by public and private facility type. Legend: Facilities include GSVM Medical College (public hospital) and Paliwal Diagnostics (private diagnostic laboratory). Numbers at bottom of each bar represent the sample size in each facility and period.

A significant increase in measles seroprevalence was observed following the SIA among children attending a public hospital but not for those attending private facilities in Kanpur Nagar District (Figure 3, Supplementary Table S6). Younger children whose specimens were tested at a public facility in Kanpur Nagar District had significantly lower rubella seroprevalence prior to the SIA compared to those attending a private hospital, though this difference was not observed following the SIA or for older children at either time point.

In Palghar District, the originating facilities included subdistrict hospitals, rural hospitals, and primary health centers. For younger children, both measles and rubella seroprevalence were highest among those attending rural hospitals compared to subdistrict hospitals or primary health centers. There was no difference in measles or rubella seroprevalence by facility type among older children (Supplementary Table S7).

### Trends in measles and rubella seroprevalence comparing residual and community serosurveys

**Figure 4.**
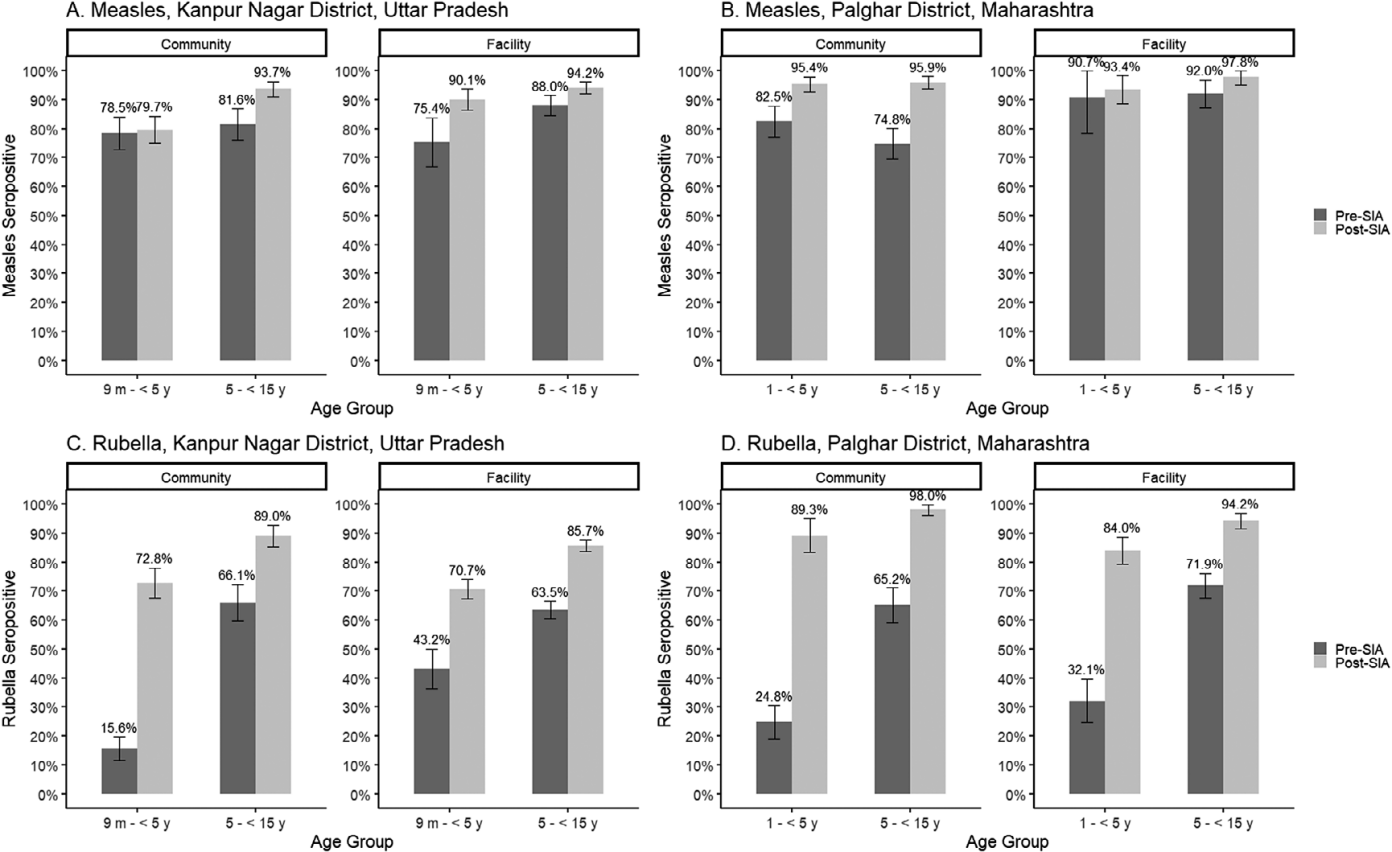
Measles and rubella seroprevalence before and after the measles-rubella supplemental immunization activity among children 9 months to <15 years, by specimen source. Legend: In Palghar District, Maharashtra, all analyses are restricted to children 1 year and older due to lack of facility specimens for children 9 months – 1 year. For community analyses, age is age at time of survey and estimates are survey weighted. Facility samples age-standardized to pre-SIA community age distribution.

Although these studies were not designed to test differences in seroprevalence between the community and facility-based specimens, descriptive results of the changes in measles and rubella seroprevalence following the SIA provide insights into the comparability of the estimates derived from the two specimen sources following age standardization to align with the age distribution in the community-based survey. No change in measles seroprevalence was observed following the SIA among younger children in the community-based serosurvey (pre-SIA 78.5 [72.8, 84.1] vs. post-SIA 79.7 [75.0, 84.4]; Figure 4, Supplementary Table S8), however, seroprevalence increased in the younger children attending the facilities in Kanpur Nagar District (seroprevalence [95% CI]: pre-SIA 75.4 [66.9, 83.8] vs. post-SIA 90.1 [86.4, 93.8]). The measles seroprevalence post-SIA among younger children enrolled in the community-based serosurvey was significantly lower than that among children attending the facilities. For the older age group in Kanpur Nagar District, the increase after the SIA was observed using either specimen source, and there was no difference in the estimates by specimen source before or after the SIA. In Palghar District, measles seroprevalence increased following the SIA among younger children enrolled in the community survey (pre-SIA 82.5 [77.3, 87.8] vs. post-SIA 95.4 [92.8, 98.0]), but not among those attending the facility (pre-SIA 90.7 [78.6, 100] vs. post-SIA 93.4 [88.6, 98.3]). An increase in measles seroprevalence following the SIA was observed for older children enrolled in the community survey (pre-SIA 74.8 [69.6, 80.1] vs. post-SIA 95.9 [93.7, 98.1]), but this was not significantly different for those attending the facility (pre-SIA 92.0 [87.2, 96.9] vs. post-SIA 97.8 [95.1, 100]). For both age groups, there was no difference by specimen source in the post-SIA seroprevalence.

Rubella seroprevalence was significantly lower prior to the SIA among younger children in the community compared to those attending the facilities in Kanpur Nagar District (15.6 [11.5, 19.7] versus 43.2 [36.3, 50.0]); however, no difference was observed following the SIA for either age group. No significant differences in rubella seroprevalence were observed in Palghar District when comparing residual and community serosurveys for either age group or timepoint, and similar trends of increasing seroprevalence were observed in both serosurveys.

### Sensitivity analyses to better align the geographic distribution of community and residual specimens

**Figure 5.**
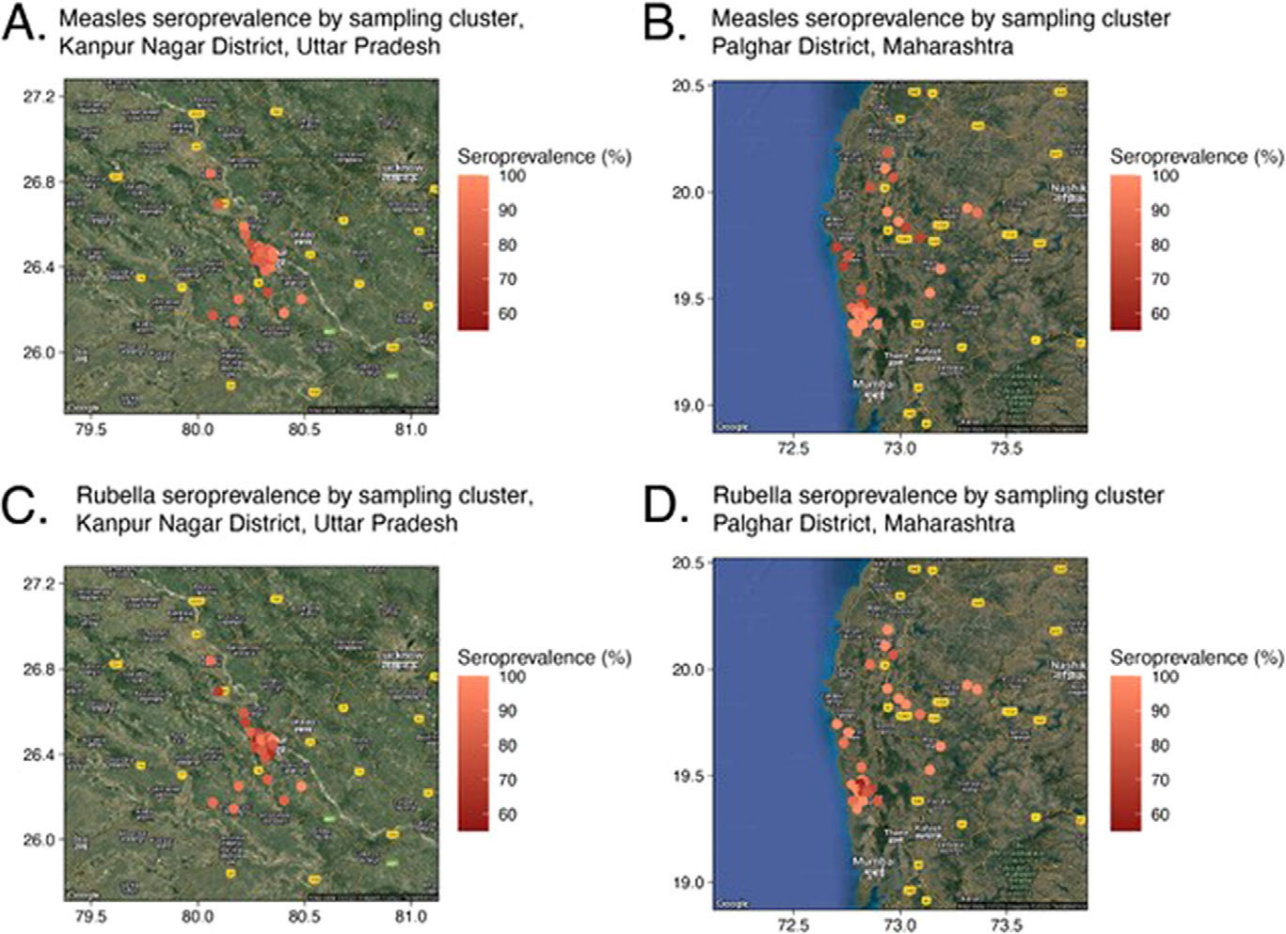
Seroprevalence by sampling cluster from community-based serosurvey in Kanpur Nagar District, Uttar Pradesh and Palghar District, Maharashtra.

In Kanpur Nagar District, both facilities where residual specimens were collected were located within the Kanpur metropolitan area, but 40% of the survey clusters were located outside the metro area (Figure 1). In contrast, in Palghar District, 43% of the 60 survey clusters in the community-based serosurvey were in the highly urban area of Vasai-Virar because clusters were selected based on probability proportional to size. However, few residual specimens collected from health facilities located in this area were received at Hind Laboratories. Sensitivity analyses restricting the community-based serosurveys to clusters that were more closely aligned with where the residual specimens originated were conducted to recalculate measles and rubella seroprevalence. However, little difference was observed when comparing the original estimates to the restricted estimates (Supplementary Tables S9 and S10), and the changes did not result in estimates closer to those from the residual serosurvey. The Moran’s I statistic for measles and rubella seroprevalence results by cluster based on the community-based sample in Palghar and Kanpur Nagar Districts were not statistically significant (Supplementary Table S11), demonstrating there was no evidence of spatial clustering by seroprevalence (Figure 5, Supplementary Figure S1). Due to the lack of spatial variation, spatial-weighted seroprevalence estimates from the facility-based samples were not compared to the seroprevalence estimates from the community-based samples.

## Discussion

Using residual specimens collected from children visiting health facilities and diagnostic laboratories, we documented increases in measles and rubella seroprevalence in two districts in India following the MR SIA, although with some inconsistencies and variability by type of facility. In Kanpur Nagar District, a significantly higher measles seroprevalence was observed following the SIA for both age groups using residual specimens, but no significant change was observed in the younger age group using community specimens. However, the opposite was observed in Palghar District, with no change following the SIA for either age group using residual specimens but a significant increase for both age groups using the community specimens. Older children in Palghar District attending the facility had significantly higher measles seroprevalence prior to the SIA compared to those enrolled in the community survey. The reasons for these differences in measles seroprevalence are unclear but did not appear to be due to spatial variation in the specimen source. An increase in rubella seroprevalence following the SIA was observed using both specimen sources and districts, and no differences by specimen source were observed in the post-SIA rubella seroprevalence in either district or age group.

The few prior studies comparing residual and community-based were conducted in high-income settings [12–14]. Our study was conducted in a lower-middle-income setting and obtained pediatric residual specimens from different types of public and private facilities. Which individuals are more likely to attend certain health facilities, and how the characteristics of those individuals are correlated with seroprevalence (e.g., socioeconomic status, vaccine access), likely varies by setting and by antigen (VPD vs. non-VPD). These factors may in turn influence how well residual specimens represent the community. Unlike prior studies, we estimated seroprevalence at two timepoints and observed the residual versus community seroprevalence estimates varied by timepoint and antigen.

Rubella-containing vaccine was only available in the private sector in India prior to the recent MR SIA, commonly administered as measles-mumps-rubella vaccine according to the recommendations of the Indian Association of Paediatrics [20]. The rubella seroprevalence findings in Kanpur Nagar District reflect this, with significantly higher seroprevalence prior to the SIA among younger children seen at private versus public facilities, likely due to differential access to the rubella-containing vaccine, but no difference among older children in whom seropositivity is primarily due to natural infection. The National Family Health Survey (NFHS-5, 2020–2021) estimated 3.6% of children 12 – 23 months living in Kanpur Nagar District received most of their vaccinations in a private health facility [21, 22]. Community-based rubella seroprevalence for younger children is more similar to that from residual specimens collected at the public facility, reflecting the fact that most children in the district received vaccines in public settings and may not have had access to rubella-containing vaccine prior to the SIA. In Kanpur Nagar District, the impact of the SIA was seen most clearly among children who attended the public facilities, with significant increases in seroprevalence for both antigens and age groups. In the private setting, a significant increase was only observed for rubella among older children. This may reflect higher pre-SIA measles and rubella seroprevalence for younger children in private facilities, possibly related to increased access to vaccines associated with characteristics of individuals attending private facilities or lower uptake of the SIA among children who attended private facilities as observed in a qualitative study in Kerala [23].

Community-based serosurveys are commonly considered the “gold standard” for estimating seroprevalence as they are designed to be representative of the population. However, the planning and fieldwork for these surveys are time-consuming and costly, typically requiring multiple survey teams and vehicles [24]. Community-based serosurveys are also subject to biases in the sampling frame due to incorrect or outdated population estimates and unavailability or non-response, especially for blood collection. In contrast, residual specimens utilize previously collected blood specimens, so the staffing, cost, and transportation requirements are significantly reduced compared to community-based serosurvey. This enables collection of specimens over a longer period or at multiple timepoints to assess trends. Facility-based specimens often allow for a wider age range of specimens, including ages difficult to capture in community-based surveys, such as school-aged children or working adults who may be unavailable when the survey is conducted. However, the primary concern with residual specimens is the lack of representativeness of the target population due to differences in geographic distribution and health care access. Limited metadata is available for residual specimens, which makes it difficult to explore the characteristics of individuals attending the facility and conduct adjusted analyses.

There are limitations to this work that may impact the findings. Due to timing of the SIA, we were only able to collect a limited number of pre-SIA specimens, and the smaller sample size may have affected comparisons with post-SIA residual and community-based estimates, especially for subgroup analyses by facility type. Few metadata were available for the residual specimens, such as information on individual or household characteristics or vaccination history, which can limit the ability to target immunity gaps or inferences about the representativeness of the sample. Similarly, geographic data on where the patient resided was not available, although we had information on the origin of residual specimens received at the diagnostic laboratories. The community-based serosurvey was designed to be representative at the district level; however, residual specimens were only collected from a small number of facilities in each district. We were able to include a mix of facility types (private, public) as well as diagnostic laboratories that received specimens from different sources across the districts, although we are unable to confirm the spatial distribution of the patients. Although we related the public versus private findings in Kanpur Nagar District to private access to vaccines, we cannot confirm if patients attending the private diagnostic laboratory also received their vaccines through the private sector. However, it is unlikely the higher rubella seroprevalence in the private residual specimens would have been driven by natural exposure given the expected levels of rubella transmission in the community.

Using residual specimens from health facilities, we measured the impact of the MR SIA on measles and rubella seroprevalence, although there were some differences from what was observed using community-based estimates. Few published studies are available that directly compare community-based and residual specimen serosurveys [12–14]. While our study provides one example of this comparison in a low-and middle-income setting, additional studies are needed to further explore this comparison in different settings. Despite challenges with representativeness and limited data available, residual specimens can play an important role in estimating seroprevalence and assessing trends [11]. Establishment of sentinel facility-based surveillance would enable ongoing surveillance at low cost to monitor changes in seroprevalence over time due to changes or disruptions in the immunization programmes and potentially detect immunity gaps, which may increase the risk of outbreaks [25].

## Supporting information

Prosperi et al. supplementary material Prosperi et al. supplementary material

## Data Availability

A subset of the key anonymised individual participant data collected during the study, along with a data dictionary, is available upon request made to the corresponding author, after approval of a proposal by the study core investigators with a signed data-access agreement.

## Supplementary material

For supplementary material accompanying this paper visit http://doi.org/10.1017/S0950268824001353. click here to view supplementary material
