# Supplementary material for: Increased measles and rubella seroprevalence in children using residual blood samples from health facilities and household serosurveys after supplementary immunization activities in two districts in India
Source: Epidemiol Infect. 2024 Nov 18;152:e143. doi: 10.1017/S0950268824001353 (PMC11574605; doi:10.1017/S0950268824001353)
Supplement: Prosperi et al. supplementary material [file S0950268824001353sup001.docx]

***Epidemiology and Infection***

**Increased measles and rubella seroprevalence in children using residual blood samples from health facilities and household serosurveys after supplementary immunization activities in two districts in India**

Authors: Christine Prosperi, Alvira Z Hasan, Amy K Winter, Itta Krishna Chaaithanya, Neha R Salvi, Sandeep Sharma, Avi Kumar Bansal, Sanjay L Chauhan, Ragini N Kulkarni, Abhishek Lachyan, Poonam Gawali, Mitali Kapoor, Arpit Kumar Shrivastava, Saurabh K Chonker, Vaishali Bhatt, Ojas Kaduskar, Gururaj Rao Deshpande, Ignacio Esteban, R Sabarinathan, Velusamy Saravana Kumar, Shaun A Truelove, Muthusamy Santhosh Kumar, Jeromie W Vivian Thangaraj, Lucky Sangal, Sanjay M Mehendale, Gajanan N Sapkal, Nivedita Gupta, Kyla Hayford, William J Moss, Manoj V Murhekar

**Supplementary Tables and Figures**

**Supplementary Table S1: Health facilities where residual specimens were collected**

| **District** | **Facility** | **Type** | **Funding** | **Catchment area** | **Setting** | **Originating source of specimen** |
| --- | --- | --- | --- | --- | --- | --- |
| Palghar | Subdistrict hospital, Dahanu | Hospital | Public | Dahanu subdistrict | Majority rural | Collected onsite only |
|  | Subdistrict hospital, Kasa | Hospital | Public | Kasa subdistrict | Majority rural | Collected onsite only |
|  | Hind Dahanu | Diagnostic laboratory | Public | Dahanu and Kasa subdistrict | Majority rural | Received from other facilities + collected onsite |
|  | Hind Jawhar | Diagnostic laboratory | Public | Jawhar subdistrict | Majority rural | Received from other facilities + collected onsite |
| Kanpur Nagar | GSVM Medical College | Hospital | Public | Kanpur Nagar district and neighboring districts | Majority urban | Collected onsite only |
|  | Paliwal Diagnostics | Diagnostic laboratory | Private | Kanpur Nagar district and neighboring districts | Majority urban | Received from other facilities + collected onsite |

**Supplementary Table S2: SIA and residual specimen collection dates by site**


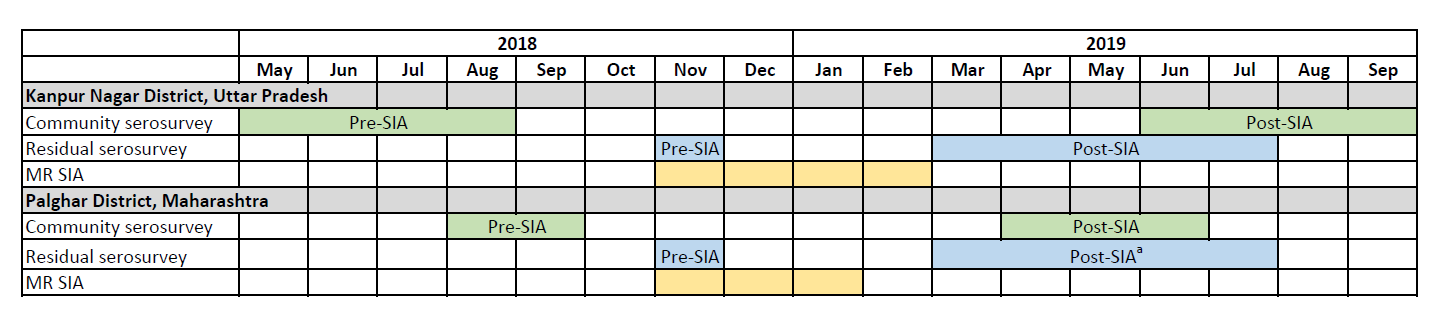


Green rows indicate community survey, blue cells indicate residual serosurvey, yellow cells indicate MR SIA.

a. Collection at the two subdistrict hospitals was stopped at the end of April 2019 due to limited number of available specimens.

**Supplementary Figure S1:** Distance-based spatial weight matrix by sampling cluster in A) Palghar District, Maharashtra and B) Kanpur Nagar District, Uttar Pradesh


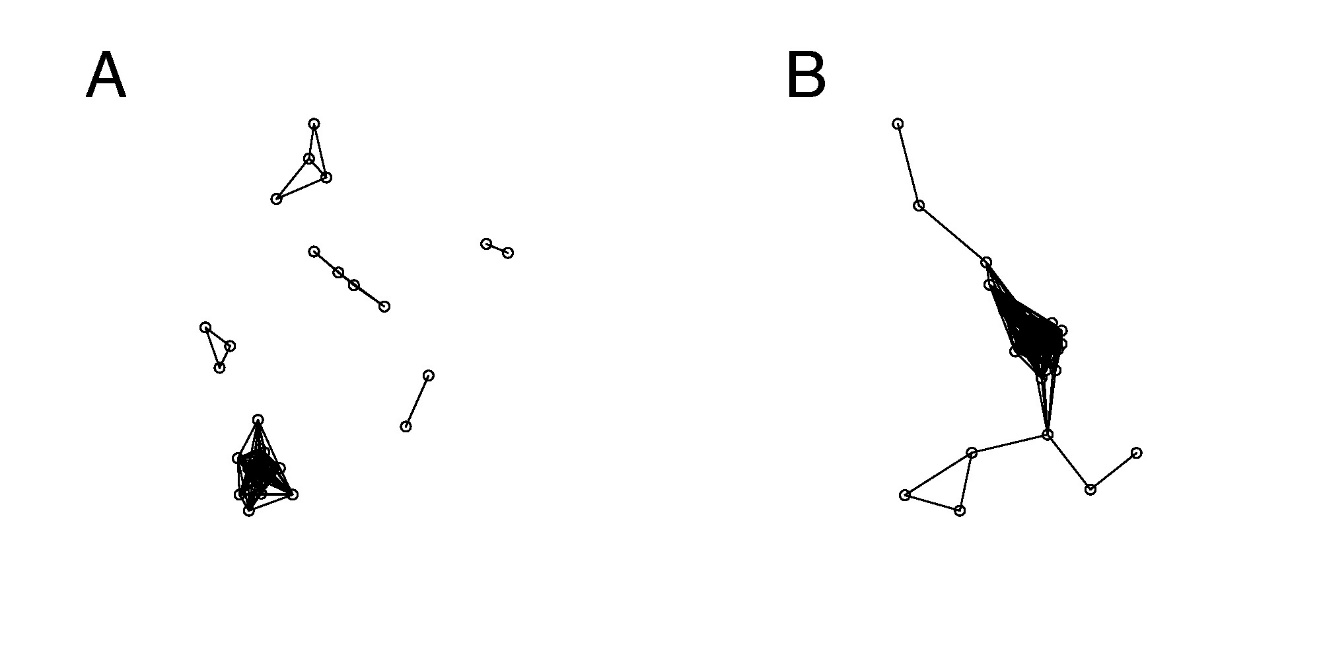


**Supplementary Table S3: Number of specimens collected by time period, age group and sex in Kanpur Nagar District, Uttar Pradesh, by public and private facility type**

|  | **Public Facility** | | **Private Facility** | |
| --- | --- | --- | --- | --- |
|  | **Pre-SIA**  **n (%)** | **Post-SIA**  **n (%)** | **Pre-SIA**  **n (%)** | **Post-SIA**  **n (%)** |
| **Total** | 166 | 655 | 232 | 705 |
| **Age group** |  |  |  |  |
| 9 m-<5 y | 43 (25.9) | 192 (29.3) | 63 (27.2) | 289 (41.0) |
| 5-<15 y | 123 (74.1) | 463 (70.7) | 169 (72.8) | 416 (59.0) |
| **Sex** |  |  |  |  |
| Female | 71 (42.8) | 289 (44.1) | 93 (40.1) | 247 (35.0) |
| Male | 95 (57.2) | 366 (55.9) | 139 (59.9) | 458 (65.0) |

Restricted to specimens 9 months and older with available EIA results.

**Supplementary Table S4: Measles and rubella seroprevalence before and after the MR SIA in each district among children 9 months to <15 years**

| **Characteristics** | **Kanpur Nagar District, Uttar Pradesh** | | | **Palghar District, Maharashtra** | | |
| --- | --- | --- | --- | --- | --- | --- |
|  | **Pre-SIA** | **Post-SIA** | **p-value^a^** | **Pre-SIA** | **Post-SIA** | **p-value^a^** |
|  | % (95% CI) | % (95% CI) |  | % (95% CI) | % (95% CI) |  |
| **Measles** | | | | | | |
| **Age group** |  |  |  |  |  |  |
| 9 m-<5 y | 73.6 (65.2, 82.0) | 89.8 (87.1, 92.5) | **< 0.001** | 90.6 (82.7, 98.4) | 92.6 (89.7, 95.5) | 0.60 |
| 5-<15 y | 88.7 (85.1, 92.3) | 94.7 (93.2, 96.1) | **< 0.001** | 93.8 (90.0, 97.5) | 98.1 (96.9, 99.3) | **0.004** |
| **Rubella** | | | | | | |
| **Age group** |  |  |  |  |  |  |
| 9 m-<5 y | 39.6 (30.3, 48.9) | 70.7 (66.6, 74.8) | **< 0.001** | 34.0 (21.2, 46.7) | 82.6 (78.4, 86.8) | **< 0.001** |
| 5-<15 y | 66.4 (61.0, 71.9) | 86.3 (84.1, 88.6) | **< 0.001** | 75.6 (69.0, 82.3) | 94.3 (92.3, 96.3) | **< 0.001** |

a. P-values from logistic regression model run comparing post versus pre-SIA adjusted for age in years. Bold indicates p-value < 0.012 (Bonferroni corrected p-value for within site and within antigen comparisons [N=4]).

**Supplementary Table S5: Measles and rubella seroprevalence before and after the MR SIA in each district among children 9 months to <15 years, by sex**

|  | **Kanpur Nagar District, Uttar Pradesh** | | | | **Palghar District, Maharashtra** | | | |
| --- | --- | --- | --- | --- | --- | --- | --- | --- |
|  | **Female** | | **Male** | | **Female** | | **Male** | |
|  | **Pre-SIA**  **(N=164)** | **Post-SIA**  **(N=536)** | **Pre-SIA**  **(N=234)** | **Post-SIA**  **(N=824)** | **Pre-SIA**  **(N=104)** | **Post-SIA**  **(N=371)** | **Pre-SIA**  **(N=109)** | **Post-SIA**  **(N=467)** |
|  | % (95% CI) | % (95% CI) | % (95% CI) | % (95% CI) | % (95% CI) | % (95% CI) | % (95% CI) | % (95% CI) |
| **Measles** |  |  |  |  |  |  |  |  |
| **Age group** |  |  |  |  |  |  |  |  |
| 9 m-<5 y | 77.3  (64.9, 89.7) | 91.6  (87.3, 95.8) | 71.0  (59.7, 82.3) | 88.9  (85.4, 92.4) | 91.7  (80.6, 100) | 93.9  (89.9, 98.0) | 89.7  (78.6, 100) | 91.6  (87.5, 95.7) |
| 5-<15 y | 89.2  (83.6, 94.7) | 95.9  (93.9, 98.0) | 88.4  (83.6, 93.2) | 93.7  (91.6, 95.8) | 93.8  (88.4, 99.1) | 97.5 (95.5, 99.5) | 93.8  (88.4, 99.1) | 98.6  (97.3, 100) |
| **Rubella** |  |  |  |  |  |  |  |  |
| **Age group** |  |  |  |  |  |  |  |  |
| 9 m-<5 y | 40.9  (26.4, 55.4) | 70.5  (63.5, 77.4) | 38.7  (26.6, 50.8) | 70.8  (65.8, 75.8) | 29.2  (11.0, 47.4) | 84.8  (78.7, 91.0) | 37.9  (20.3, 55.6) | 80.9  (75.1, 86.7) |
| 5-<15 y | 63.3  (54.7, 72.0) | 87.3  (83.9, 90.7) | 68.6  (61.7, 75.5) | 85.7  (82.6, 88.7) | 77.5  (68.3, 86.7) | 93.7  (90.6, 96.8) | 73.8  (64.1, 83.4) | 94.8  (92.2, 97.4) |

No residual specimens collected from children between 9 months to 1 year in Palghar District.

**Supplementary Table S6: Measles and rubella seroprevalence before and after the MR SIA in Kanpur Nagar District, Uttar Pradesh among children 9 months to <15 years, by facility type**

| **Characteristics** | **Public** | | | **Private** | | | **Public vs. Private** | |
| --- | --- | --- | --- | --- | --- | --- | --- | --- |
|  | **Pre-SIA**  **(N=166)** | **Post-SIA**  **(N=655)** | **Post vs Pre p-value^a^** | **Pre-SIA**  **(N=232)** | **Post-SIA**  **(N=705)** | **Post vs Pre p-value^a^** | **Pre-SIA**  **p-value^a^** | **Post-SIA**  **p-value^a^** |
|  | % (95% CI) | % (95% CI) |  | % (95% CI) | % (95% CI) |  |  |  |
| **Measles** | | | | | | | | |
| **Age group** |  |  |  |  |  |  |  |  |
| 9 m-<5 y | 65.1 (50.9, 79.4) | 90.6 (86.5, 94.7) | **< 0.001** | 79.4 (69.4, 89.4) | 89.3 (85.7, 92.8) | 0.03 | 0.11 | 0.88 |
| 5-<15 y | 89.4 (84.0, 94.9) | 97.8 (96.5, 99.2) | **< 0.001** | 88.2 (83.3, 93.0) | 91.1 (88.4, 93.8) | 0.23 | 0.83 | **< 0.001** |
| **Rubella** | | | | | | | | |
| **Age group** |  |  |  |  |  |  |  |  |
| 9 m-<5 y | 20.9  (8.8, 33.1) | 72.9  (66.6, 79.2) | **< 0.001** | 52.4  (40.0, 64.7) | 69.2  (63.9, 74.5) | 0.01 | **0.003** | 0.59 |
| 5-<15 y | 65.0  (56.6, 73.5) | 88.8  (85.9, 91.6) | **< 0.001** | 67.5  (60.4, 74.5) | 83.7  (80.1, 87.2) | **< 0.001** | 0.27 | 0.12 |

1. P-value from logistic regression model adjusted for age in years. Bold indicates p < 0.006 (Bonferroni corrected p-value for within antigen comparisons [N=8]).

Analyses stratified by public versus private facility type in Kanpur Nagar District demonstrated rubella seroprevalence significantly increased following the SIA in both age groups and facility types (Figure 2, Supplementary Table S6). However, prior to the SIA, significantly higher rubella seroprevalence was observed in younger children at the private facility than at the public facility (52.4 [40.0, 64.7] vs. 20.9 [8.8, 33.1]; Figure 2, Supplementary Table S6) but no differences were observed following the SIA for this age group. No difference in rubella seroprevalence was observed by facility type among older children at either time point. Measles seroprevalence increased following the SIA in both age groups among children attending the public facility but only marginally among the younger children for those attending private facilities (Figure 2, Supplementary Table S6). No difference in measles seroprevalence was observed by facility type prior to the SIA but seroprevalence after the SIA was significantly higher among older children attending the public facility compared to the private facility (97.8 [96.5, 99.2] vs. 91.1 [88.4, 93.8]).

**Supplementary Table S7: Measles and rubella seroprevalence after the MR SIA, by originating health facility type among children presenting to facilities in Palghar District, Maharashtra**

|  | **Subdistrict Hospital (N=366)** | **Rural Hospital**  **(N=279)** | **Primary Health Center**  **(N=193)** | **p-value^a^** |
| --- | --- | --- | --- | --- |
|  | % (95% CI) | % (95% CI) | % (95% CI) |  |
| **Measles** |  |  |  |  |
| **Age group** |  |  |  |  |
| 1-<5 y | 91.6  (87.6, 95.7) | 96.7  (93.0, 100.4) | 87.8  (77.8, 97.8) | 0.09 |
| 5-<15 y | 97.3  (95.0, 99.6) | 97.9  (95.8, 99.9) | 99.3  (98.1, 100.6) | 0.37 |
| **Rubella** |  |  |  |  |
| **Age group** |  |  |  |  |
| 1-<5 y | 79.3  (73.4, 85.3) | 91.1  (85.2, 97.0) | 78.0  (65.4, 90.7) | 0.03 |
| 5-<15 y | 92.0  (88.1, 95.9) | 94.2  (90.8, 97.5) | 97.4  (94.8, 99.9) | 0.14 |

1. P-value from logistic regression model adjusted for age in years.

Due to small sample size prior to the SIA in Palghar District, most of which originated from subdistrict hospitals (55.9%, Table 1), analyses by facility type were limited to the post-SIA specimens.

**Supplementary Table S8: Measles and rubella seroprevalence before and after the MR SIA among children 9 months to <15 years, by specimen source**

1. **Kanpur Nagar District, Uttar Pradesh**

|  | **Kanpur Nagar District, Uttar Pradesh** | | | | | | | | | | |
| --- | --- | --- | --- | --- | --- | --- | --- | --- | --- | --- | --- |
|  | **Facility-based serosurvey** | | | | **Community-based serosurvey** | | | | **Facility vs Community** | | |
|  | **Pre-SIA** | **Post-SIA** | **Post vs Pre p-value^a^** | **Pre-SIA** | | **Post-SIA** | **Post vs Pre p-value^a^** | **Pre-SIA**  **p-value^a^** | | **Post-SIA**  **p-value^a^** |  |
|  | % (95% CI) | % (95% CI) |  | % (95% CI) | | % (95% CI) |  |  | |  |  |
| **Measles** |  |  |  |  | |  |  |  | |  |  |
| **Age group** |  |  |  |  | |  |  |  | |  |  |
| 9 m-<5 y | 75.4  (66.9, 83.8) | 90.1  (86.4, 93.8) | **< 0.001** | 78.5  (72.8, 84.1) | | 79.7  (75.0, 84.4) | 0.91 | 0.45 | | **< 0.001** |  |
| 5-<15 y | 88.0  (84.5, 91.5) | 94.2  (92.1, 96.2) | **0.003** | 81.6  (76.2, 87.0) | | 93.7  (91.1, 96.3) | **< 0.001** | 0.03 | | 0.30 |  |
| **Rubella** |  |  |  |  | |  |  |  | |  |  |
| **Age group** |  |  |  |  | |  |  |  | |  |  |
| 9 m-<5 y | 43.2  (36.3, 50.0) | 70.7  (67.4, 74.1) | **< 0.001** | 15.6  (11.5, 19.7) | | 72.8  (67.6, 78.0) | **< 0.001** | **< 0.001** | | 0.61 |  |
| 5-<15 y | 63.5  (60.6, 66.5) | 85.7  (83.7, 87.7) | **< 0.001** | 66.1  (59.9, 72.4) | | 89.0  (85.3, 92.8) | **< 0.001** | 0.90 | | 0.37 |  |

a. P-value from survey weighted logistic regression model adjusted for age in years. Bold indicate p < 0.006 (Bonferroni corrected p-value threshold for within site and within antigen comparisons [N=8]).

For community analyses, age is age at time of survey. Analyses in Palghar District were to children > 1 year due to lack of children between 9 months – 1 year in the facility survey. Facility samples age-standardized to match pre-SIA community age distribution.

1. **Palghar District, Maharashtra**

|  | **Palghar District, Maharashtra** | | | | | | | | | | |
| --- | --- | --- | --- | --- | --- | --- | --- | --- | --- | --- | --- |
|  | **Facility-based serosurvey** | | | | **Community-based serosurvey** | | | | **Facility vs Community** | | |
|  | **Pre-SIA** | **Post-SIA** | **Post vs Pre p-value^a^** | **Pre-SIA** | | **Post-SIA** | **Post vs Pre p-value^a^** | **Pre-SIA**  **p-value^a^** | | **Post-SIA**  **p-value^a^** |  |
|  | % (95% CI) | % (95% CI) |  | % (95% CI) | | % (95% CI) |  |  | |  |  |
| **Measles** |  |  |  |  | |  |  |  | |  |  |
| **Age group** |  |  |  |  | |  |  |  | |  |  |
| 1-<5 y | 90.7  (78.6, 100.0) | 93.4  (88.6, 98.3) | 0.65 | 82.5  (77.3, 87.8) | | 95.4  (92.8, 98.0) | **< 0.001** | 0.18 | | 0.51 |  |
| 5-<15 y | 92.0  (87.2, 96.9) | 97.8  (95.1, 100) | 0.02 | 74.8  (69.6, 80.1) | | 95.9  (93.7, 98.1) | **< 0.001** | **< 0.001** | | 0.06 |  |
| **Rubella** |  |  |  |  | |  |  |  | |  |  |
| **Age group** |  |  |  |  | |  |  |  | |  |  |
| 1-<5 y | 32.1  (24.5, 39.7) | 84.0  (79.3, 88.7) | **< 0.001** | 24.8  (19.0, 30.6) | | 89.3  (83.5, 95.1) | **< 0.001** | 0.13 | | 0.23 |  |
| 5-<15 y | 71.9  (67.7, 76.1) | 94.2  (91.6, 96.9) | **< 0.001** | 65.2  (59.3, 71.2) | | 98.0  (96.2, 99.8) | **< 0.001** | 0.04 | | 0.01 |  |

a. P-value from survey weighted logistic regression model adjusted for age in years. Bold indicate p < 0.006 (Bonferroni corrected p-value threshold for within site and within antigen comparisons [N=8]).

For community analyses, age is age at time of survey. Analyses in Palghar District were to children > 1 year due to lack of children between 9 months – 1 year in the facility survey. Facility samples age-standardized to match pre-SIA community age distribution.

**Supplementary Table S9: Measles and rubella seroprevalence before and after the MR SIA, by specimen source among children residing in or presenting to facilities located in urban areas in Kanpur Nagar District, Uttar Pradesh**

| **Antigen and age group** | **Facility-based serosurvey** | | **Community-based serosurvey** | | | |
| --- | --- | --- | --- | --- | --- | --- |
|  |  |  | **All survey clusters** | | **Restricted to survey clusters inside Kanpur City** | |
|  | **Pre-SIA** | **Post-SIA** | **Pre-SIA** | **Post-SIA** | **Pre-SIA** | **Post-SIA** |
|  | % (95% CI) | % (95% CI) | % (95% CI) | % (95% CI) | % (95% CI) | % (95% CI) |
| **Measles** |  |  |  |  |  |  |
| **Age group** |  |  |  |  |  |  |
| 9 m-<5 y | 75.4  (66.9, 83.8) | 90.1  (86.4, 93.8) | 78.5  (72.8, 84.1) | 79.7  (75.0, 84.4) | 72.6  (64.5, 80.7) | 77.5  (71.1, 83.9) |
| 5-<15 y | 88.0  (84.5, 91.5) | 94.2  (92.1, 96.2) | 81.6  (76.2, 87.0) | 93.7  (91.1, 96.3) | 87.1  (80.8, 93.4) | 93.4  (90.1, 96.7) |
| **Rubella** |  |  |  |  |  |  |
| **Age group** |  |  |  |  |  |  |
| 9 m-<5 y | 43.2  (36.3, 50.0) | 70.7  (67.4, 74.1) | 15.6  (11.5, 19.7) | 72.8  (67.6, 78.0) | 19.7  (13.8, 25.6) | 70.0  (63.1, 76.8) |
| 5-<15 y | 63.5  (60.6, 66.5) | 85.7  (83.7, 87.7) | 66.1  (59.9, 72.4) | 89.0  (85.3, 92.8) | 65.6  (57.3, 73.8) | 84.7  (78.4, 91.0) |

**Supplementary Table S10: Measles and rubella seroprevalence before and after the MR SIA, by specimen source among children residing in or presenting to facilities located in rural areas in Palghar District, Maharashtra**

| **Antigen and age group** | **Facility-based serosurvey** | | **Community-based serosurvey** | | | |
| --- | --- | --- | --- | --- | --- | --- |
|  |  |  | **All survey clusters** | | **Excluding survey clusters in Vasai-Virar** | |
|  | **Pre-SIA** | **Post-SIA** | **Pre-SIA** | **Post-SIA** | **Pre-SIA** | **Post-SIA** |
|  | % (95% CI) | % (95% CI) | % (95% CI) | % (95% CI) | % (95% CI) | % (95% CI) |
| **Measles** |  |  |  |  |  |  |
| **Age group** |  |  |  |  |  |  |
| 1-<5 y | 90.7  (78.6, 100.0) | 93.4  (88.6, 98.3) | 82.5  (77.3, 87.8) | 95.4  (92.8, 98.0) | 84.4  (78.7, 90.1) | 93.9  (90.0, 97.7) |
| 5-<15 y | 92.0  (87.2, 96.9) | 97.8  (95.1, 100.0) | 74.8  (69.6, 80.1) | 95.9  (93.7, 98.1) | 69.9  (62.8, 77.0) | 93.4  (89.7, 97.0) |
| **Rubella** |  |  |  |  |  |  |
| **Age group** |  |  |  |  |  |  |
| 1-<5 y | 32.1  (24.5, 39.7) | 84.0  (79.3, 88.7) | 24.8  (19.0, 30.6) | 89.3  (83.5, 95.1) | 17.9  (11.7, 24.1) | 98.0  (95.5, 100.6) |
| 5-<15 y | 71.9  (67.7, 76.1) | 94.2  (91.6, 96.9) | 65.2  (59.3, 71.2) | 98.0  (96.2, 99.8) | 59.2  (51.7, 66.7) | 98.1  (95.8, 100) |

a. Younger age group reflect 1-<5y for Palghar District, Maharashtra for both facility and community since there were no children < 1 year in the facility-based serosurvey.

| Supplementary Table S11. Spatial analysis of community-based serosurvey measles and rubella seroprevalence, Moran's I statistic results | | | | |
| --- | --- | --- | --- | --- |
|  | **Palghar, Maharashtra** | | **Kanpur Nagar, Uttar Pradesh** | |
|  | **Measles** | **Rubella** | **Measles** | **Rubella** |
| Moran's Index, (SD) | 0.21 (1.71) | 0.14 (1.27) | -0.06 (-0.22) | 0.05 (0.87) |
| Expected Index | -0.03 | -0.03 | -0.03 | -0.03 |
| Variance | 0.02 | 0.019 | 0.01 | 0.01 |
| *p-value (Monte Carlo simulation, 1000 permutations)* | 0.058 | 0.116 | 0.546 | 0.162 |

A Moran’s I statistic above 0 with a p-value <0.05 indicates a spatial correlation pattern between cluster seroprevalence.

**Supplementary Appendix I: Methods**

**Background on setting and residual specimen collection procedures**

**Palghar District, Maharashtra**

At the time of designing the study (2018), Palghar District had a population of 2,990,116. Only one block out of eight in the district is urban (Vasai-Virar) which is where the majority of the population lives. There are three subdistrict hospitals (SDHs) in Dahanu and Jawhar blocks and no district hospital. A substantial proportion of the population uses private sector hospitals and clinics, especially in urban areas. In 2018 there were 90-95 private hospitals in the district: 48 rural and 25 urban private hospitals in the area catered by SDH Dahanu, 12 rural private hospitals in the area catered by SDH Kasa and 7-8 rural private hospitals in the area catered by SDH Jawhar. Blood is collected at all facilities and may be tested onsite or sent to a diagnostic center.

HLL Lifecare Ltd. (or Hind Lab) signed an agreement with the Ministry of Health and Family Welfare, Government of Maharashtra in 2018 to provide diagnostic laboratory services for state-run health facilities. In Palghar District, there are two Hind lab centers located in Dahanu and Jawhar, which provides diagnostic services for tests that cannot be conducted in SDHs, rural hospitals (RH) and PHCs. For example, the SDH conducts basic tests for antenatal care patients, like renal function testing and blood group typing, and sends specimens for all other tests to Hind (like C-reactive protein and thyroid function tests). Approximately 8000 plasma and 5000 serum specimens are processed at the two Hind laboratories each month, 65-75% of which are from PHCs, 17-23% from RHs, and 2-17% from SDH.

For this project the three subdistrict public hospitals and three diagnostic laboratories (two private and one public) were identified as potential locations for specimen collection. One private facility refused and one other did not respond to the request but the public diagnostic laboratory (Hind Labs) agreed to participate. One subdistrict hospital (Jawhar) was excluded due to distance to the Model Rural Health Research Unit (MRHRU) laboratory where the specimens were processed and because most of their specimens were sent to Hind Lab Jawhar.

To inform the design, we collected data from the health facilities on the number of specimens collected or received at Hind Lab each month by specimen type (plasma or serum) and age group to understand patient flow and approximate how long it will take to reach the target sample size. This information was also important to understand how specimens moved from the SDH to the Hind laboratories. We estimated approximately 30-60 serum specimens were received monthly for each pediatric age group (0-4 years and 5-15 years) at each of the two Hind Labs.

**Kanpur Nagar District, Uttar Pradesh**

At the time of designing the study (2018), Kanpur Nagar District had a population of 4,581,000 with 66% of the population living in urban areas and 34% of population living in rural areas (<https://kanpurnagar.nic.in/demography/>).

The Lala Lajpat Rai Hospital, attached to the GSVM Medical College in Kanpur Nagar, is a 1055 bed tertiary care hospital with the laboratory under the pathology department serving over 2000 outpatients per day (<http://gsvmmedicalcollege.com/hospital-overview/>). The catchment area includes Kanpur Nagar District and neighboring districts. In this catchment area, private clinic use is high and related to ability to pay and location.

Residual blood specimens were collected from the Medical College laboratory which conducted microbiology and hematology testing from the routine OPD and internal medicine departments. Some specimens from the serology, orthopedic and gynecology departments may also be tested in the lab for specific tests, although gynecology and serology departments typically test their specimens separately. Sera are typically tested for biochemistry tests then discarded after testing is complete. Variables routinely collected for residual specimens from GSVM Medical College included age, sex, date of collection, patient location (inpatient/outpatient) and if patient was seen for antenatal care.

Paliwal Diagnostic Laboratory is a private diagnostic laboratory that conducts testing for over 60 collection centers in Kanpur Nagar District and neighboring districts. Specimens are collected at the collection centers, transported to Paliwal Main Laboratory each day and sera are tested for biochemistry, microbiology and other clinical tests. Specimens are kept at 4C for 48-72 hours days and then discarded. Variables collected for residual specimens from Paliwal Diagnostic Lab included age, sex, date of collection and collection center.

Based on discussions with facility staff we estimated Paliwal Diagnostics collected between 2500-3000 specimens per day and GSVM Medical College collected between 1500-2000 specimens per day.

**Specimen testing procedures**

Four samples per plate were randomly selected in duplicate to monitor intra-plate variability. Every 20^th^ specimen per plate was retested to assess interplate variability. Samples with measles IgG of ≥200 mIU/mL were considered seropositive, <150 mIU/mL were considered seronegative, and between ≥ 150 to <200 mIU/mL were considered equivocal. The corresponding thresholds for rubella IgG were ≥11, <8 and ≥ 8 to <11 IU/mL, respectively. Equivocal samples were retested in duplicate using the same assay. Of the three qualitative results, the most frequent was selected as the final qualitative result. Samples determined to be equivocal after retesting were treated as positive in analyses.
